# Improvement of muscle strength in specific muscular regions in nusinersen-treated adult patients with 5q-spinal muscular atrophy

**DOI:** 10.1038/s41598-023-31617-5

**Published:** 2023-04-17

**Authors:** Olivia Schreiber-Katz, Hannah Alexandra Siegler, Gary Wieselmann, Mareike Kumpe, Gresa Ranxha, Susanne Petri, Alma Osmanovic

**Affiliations:** 1 Department of Neurology, Hannover Medical School, 30625 Hannover, Germany; 2 Essen Center for Rare Diseases (EZSE), University Hospital Essen, Hufelandstrasse 55, 45147 Essen, Germany

**Keywords:** Neuromuscular disease, Motor neuron disease

## Abstract

Real-world data have shown mild improvement of overall motor function in adult patients treated with nusinersen, the first approved therapy for 5q-spinal muscular atrophy (SMA). However, knowledge about preferably targeted muscle functions is sparse. The aim of this study was to evaluate strength of distinct muscles and body regions in adult SMA patients in the early course of nusinersen therapy. 72 muscles of 15 patients were tested on the Medical Research Council (MRC) 0–10 scale (translated into MRC %) from nusinersen start to 14 months of treatment. The whole body muscular strength improved slightly or remained stable in 80% of SMA patients with a median improvement of + 2%. However, relevant increases of muscle strength of distinct regions were identified in the proximal upper limbs and shoulder girdle (median + 8%) and in muscle groups with a preserved function pre-treatment, even in more advanced diseased SMA patients. MRC grading was additionally performed in seven patients enrolled during ongoing treatment. Here, further improvement of muscle strength until month 18–26 was seen with the highest increases in the proximal upper and lower limbs. Our findings suggest that sole evaluation of the overall muscle strength might underestimate nusinersen therapy benefits.

## Introduction

Nusinersen was the first globally approved disease-modifying treatment for 5q-associated spinal muscular atrophy (SMA), a rare autosomal recessive motor neuron disease, leading to the progressive loss of muscle strength and growing disability. Responsible for the degeneration of alpha motor neurons is a lack of the full-length survival of motor neuron (SMN) protein, caused by mostly homozygous mutations in the *SMN1* gene on the short arm of chromosome five. The *SMN*2 gene, an almost identical gene, exists in a variable amount of copies, but can only partly rescue the SMA phenotype. The consequence is a broad spectrum of muscle weakness, commonly classified into four SMA subtypes (SMA types 1–4), depending on the best reached motor milestone and age of onset^1,2^.

Nusinersen, an antisense oligonucleotide, promotes an increase of SMN protein by modifying *SMN2* pre-mRNA splicing. It was approved for all SMA types and ages based on two pivotal studies performed in children under the age of 12 years. Presenting a gain of motor function they revealed the ability of weak muscles to regain their strength^3,4^. However, evaluating treatment responses in adult SMA patients with variable muscle weakness and irreversible muscle atrophy and fibrosis due to longer disease durations is more challenging. Recently, efficacy of nusinersen in adult SMA patients has been repeatedly demonstrated in functional motor outcome measures obtained in real-world settings, but treatment responses seem to differ substantially between subtypes as well as individuals^5–11^.

Differentially severe affection of certain muscles and muscle groups has been discussed to contribute to the individual responses^12^, which might be missed by commonly performed outcome measures. Their focus is on evaluating general muscle strength rather than comparing efficacy of a treatment on distinct muscles or muscle regions. Hence, they are less sensitive to smaller changes in strength and may therefore not fully address individuality of treatment responses^13–17^. Currently, there are no general criteria on when to hold back, switch or terminate therapy^18^, but with an alternative therapy on the market, treatment non-responder must be identified as early as possible to prevent further disease progression^19^.

This study’s aim was to investigate nusinersen treatment responses on distinct muscles and muscle groups by extensive manual muscle strength testing.

## Methods

### Study design and participants

Between June 2018 and March 2020, all adult 5q-SMA patients treated with nusinersen at the Hannover Medical School were enrolled in a single center observational cohort study, before or during their ongoing treatment. Nusinersen treatment was applied intrathecally according to the recommended scheme, which consists of four loading doses with 12 mg at days 0, 14, 28 and 63 followed by maintenance dosing every four months^2^. Intrathecal injections were performed via either conventional lumbar puncture or computed tomography-guided lumbar puncture in those patients with severe scoliosis^20^. At study enrolment, clinical and demographic data was obtained (Table 1). Ambulatory state was defined as the ability to walk ≥ 10 m without assistance or the use of a device, such as a cane or a walker^21^.

**Table 1:** Baseline demographics of enrolled patients, divided in patients who were enrolled treatment-naïve and during ongoing treatment.

	All (n = 22)	Treatment-naïve (n = 15)	Later enrolled (n = 7)
Sex			
Female—n (%)	9 (41%)	6 (40%)	3 (43%)
Male—n (%)	13 (59%)	9 (60%)	4 (57%)
Age at symptom onset—years, median (range)	2 (0.5–47.2)	3 (0.5–47.2)	1 (0.6–8.0)
Age at treatment initiation—years, median (range)	34 (19–64)	34 (19–64)	37 (20–63)
SMA type—n (%)			
2	9 (41%)	5 (33%)	4 (57%)
3a	4 (18%)	2 (13%)	2 (29%)
3b	8 (36%)	7 (47%)	1 (14%)
4	1 (5%)	1 (7%)	0 (0%)
*SMN2* copy number—n (%)			
2	2 (9%)	0 (0%)	2 (29%)
3	8 (36%)	5 (33%)	3 (43%)
4	9 (41%)	8 (53%)	1 (14%)
≥ 5	3 (14%)	2 (13%)	1 (14%)
Ambulatory status			
Non-ambulant—n (%)	12 (55%)	7 (47%)	5 (71%)
Ambulant—n (%)	10 (45%)	8 (53%)	2 (29%)
Scoliosis	9 (41%)	5 (33%)	4 (57%)
NIV—n (%)	6 (27%)	4 (27%)	2 (29%)
PEG—n (%)	2 (9%)	1 (7%)	1 (14%)

*m* module [median, range], *n* number, *NIV* non-invasive ventilation, *PEG* percutaneous endoscopic gastrostomy, *SMA* spinal muscular atrophy, *SMN2* survival of motor neuron 2 (gene), *y* years.

Muscle strength testing was performed at every treatment visit by medical students: author pairs MK and GR or GW and HAS. Before study initiation the examiners were given full instructions and training on performing muscle strength testing to ensure uniform testing technique. During enrolment of the first patients practical performance on patients were supervised by a trained neurologist. Raters were not blinded to previous results. Patients with less than one year observation duration were excluded from further analysis.

### Muscle strength measures

Manual muscle testing (MMT), an established and easily performed tool in clinical routine, typically measures muscle strength according to the six-point Medical Research Council (MRC) score. A score of zero defines a plegic muscle, whereas five indicates normal muscle strength. MMT has been found feasible, reliable and sensitive to compare different muscle groups and detect changes in a slowly progressive disease. It was previously used across different SMA types in natural history cohorts as well as in three nusinersen treatment studies^8–10,22–26^. To increase sensitivity in a short term follow-up period, the MRC has been modified to a zero to ten scale (MRC 0–10), as described in supplementary Table S1^27–29^.

Thirty-seven muscle functions, in total 72 muscles (21 for the upper limb and 14 for the lower limb, bilaterally, along with neck flexion and extension), were included in the MMT (list of assessed muscle functions in supplementary Table S2). In limbs with contractures, testing positions were adapted and grading was performed without evaluating range of motion, if feasible.

After the examination of single muscle groups, all assessed muscles were summarized into an overall score (total MRC %) and then subdivided into subdomains (supplementary Table S2)^23–25^. MRC scores were transformed into a corresponding percentage value to account for missing data, e.g. subject to injuries, named either MRC % (for individual muscle functions or subdomains of assessed muscles) or total MRC % (sum of all assessed muscles). MRC % was calculated as follows^10,30^:

$$ {\text{MRC }}\% = \left( {{\text{Score achieved }} \times {1}00} \right)/\left( {{\text{number of muscles tested }} \times { 1}0} \right). $$

To account for interrater- and test–retest differences, short-term re-assessments of MMT were performed during loading dosing (days 0, 14 and 28) and intraclass correlation coefficients (ICC) along with maximum observed variability of mean MRC % were calculated for total MRC %, MRC % of subdomains and single muscle functions. Missing single muscle strength grades were not replaced as imputation could have falsified interpretation of reliability for ICC calculation. Cases with completely missing MMT datasets at days 14 or 28 were excluded from ICC analysis (n = 3). The maximum mean deviation between the three time points ranged from 1.7% to 3.3% in total MRC % and all defined subdomains with excellent ICCs (results and ICC calculation in supplementary Table S3). For best data accuracy and comprehensibility of observations, a change in total MRC % and the subdomains above the cut-off of ≥ 5% was defined as an evident change of motor function (further referred to as relevant incline or decline, respectively). On the MRC 0–10 scale this would correspond to an improvement of a half grade of one muscle function—for instance, an improvement of 5% from grade three towards grade four could mean an increased range of motion of a muscle against gravity. Reliability varied distinctly between single muscle functions with scores from poor to excellent and maximum mean MRC % deviations of 0.4–10.0%. Therefore, an evident change of a single muscle function was defined as ≥ 5% and higher than its maximum mean MRC % deviation. Changes within these borders were referred to as stable motor function.

### Protocol approvals and registrations

The study was conducted in accordance with the Declaration of Helsinki, and approved by the Medical Ethics Committee of Hannover Medical School (No. 6269). Informed consent was obtained from all subjects involved in the study.

### Statistics

Statistical analyses were performed using IBM® Statistical Software Package of Social Science (SPSS® 156, 157 Chicago, IL, USA) version 26.0 and illustrations were made using GraphPad Prism version 9.10 for Windows (GraphPad Software, San Diego, California USA). Descriptive statistics are presented as percent (%), median and range, mean and standard deviation.

Spearman’s Rank correlation (two-tailed) was carried out to compare the relationship of total MRC % to physical characteristics (SMA type, *SMN2* copy number) pre-treatment.

Mann–Whitney-U test was used to analyze possible differences in total MRC % pre-treatment between ambulatory and non-ambulatory patients.

Due to the ongoing severe acute respiratory syndrome coronavirus 2 (SARS-CoV-2) pandemic, in five patients motor testing was omitted at month 14 to reduce personal contact in line with current regulations in our hospital. Datasets were replaced with values obtained at month 10 according to the “last observation carried forward” method^31^. Considering the slow progression of SMA and the potential cumulating effect of nusinersen over time, we deemed this method applicable, in particular with the intention not to overestimate treatment effects. Significance level was set at p ≤ 0.05 (two-tailed).

## Results

### Study population and adjustments to missing data

A total of 29 patients were enrolled in this study. Missing the required nusinersen treatment duration of one year, 7 patients were excluded from further analysis (in three cases due to treatment termination). Of the remaining 22 patients (9 females, 13 males), 7 were enrolled at variable time points of their ongoing therapy and 15 before nusinersen treatment (Table 1). In these 15 cases, 95 of 105 possible MMT datasets were acquired and analysed during the observation period of 14 months of nusinersen treatment. Five complete datasets of muscle strength testing were missing during loading dosing (three at day 14, two at day 28) and five MMT datasets at month 14 due to SARS-CoV-2 restrictions. The latter were replaced according to the “last observation carried forward” method to include all data available. In 5 of the 15 treatment-naïve enrolled cases grading of single muscle functions were missing (2 cases with one single missing grading at day 14, 2 cases with two at day 0 or 28 and 1 case with eight at day 28, respectively). In the later enrolled 7 cases, in 2 cases missing MMT gradings were detected (1 with four missing muscle strength gradings and 1 with seven missing gradings, both at month 10). The calculation of total MRC % and subdomains was adjusted by the number of tested muscles as described in the method section.

### Phenotypes and patterns of muscle involvement in 15 treatment-naïve patients

Fifteen enrolled patients, five SMA type 2, nine SMA type 3 patients and one SMA type 4 patient (seven non-ambulatory) aged 19–64 at therapy start were enrolled before nusinersen treatment initiation (Table 1).

**Figure 1:**
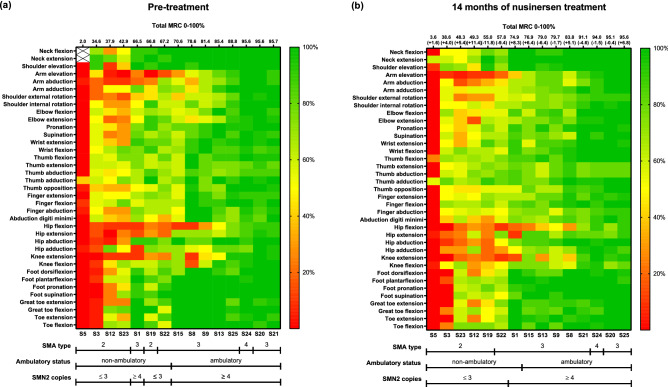
Muscle function patterns of 15 assessed patients with SMA types 2–4. Individual values are presented as the mean of both sides (with the exception of neck flexion und extension) and sorted by maximum total MRC % at baseline (**a**) or month 14 (**b**). Patients are represented by columns on the x-axis. Patients with minor muscle strength grouped on the far left. Columns on the y-axis represent the impairment pattern of specific muscle functions through the study population. Green (100%) corresponds to the absence of muscle weakness and red (0%) to plegia. Lines underneath the heatmaps indicate the corresponding distribution of SMA subtypes, ambulatory status and *SMN2* copies. (**a**) The impairment pattern at baseline shows a main involvement of proximal muscles, while distal muscle weakness was only visible in more progressed patients. (**b**) After 14 months on treatment, a trend towards regaining proximal muscle strength was seen, predominantly in the upper limbs.

MRC % ratings of 72 examined muscles of 15 SMA patients before nusinersen treatment are visualized in a heat map, ranked from the lowest to the highest total MRC % (2.0–95.7%) (Fig. 1a). A continuum of muscle strength was seen, picturing a broad disease severity spectrum with a distinct, well-known muscle weakness pattern with proximal muscles most severely affected (MRC 61% versus (vs) 80% for distal muscles) (Table 2). Proximal muscle weakness was more pronounced in the lower than the upper limbs, whereas involvement of distal muscles focused on the upper limbs and was generally less severe (individual values of subdomains at baseline and month 14 in supplementary Table S4). Looking at single muscle functions: hip flexion was identified to be affected most severely (median MRC % 20%, range 0–90%), followed by knee extension (30%, 0–100%), arm elevation (45%, 0–100%), arm abduction (55%, 5–100%), hip extension (60%, 0–95%), external and internal shoulder rotation and elbow extension, each 60% (range 0–100%). Trunk muscles, here neck flexion (median MRC % 100%, range 30–100%), neck extension (median MRC % 100%, range 60–100%) and shoulder elevation (median MRC % 100%, range 0–100%), along with muscle functions of the distal lower limb (median MRC % ranged from 85 to 100%) were among the most preserved muscles (supplementary Table S5). In contrast, distal muscles of the upper limbs showed more involvement than in the lower limbs (median MRC % ranged from 70 to 90%). Total MRC % correlated with disease characteristics such as SMA type (r = 0.807; p < 0.001, n = 15) and *SMN2* copy number (r = 0.785; p = 0.001; n = 15). Also, total MRC % discriminated well between ambulatory vs. non-ambulatory patients (median MRC total % 87% vs 43% p = 0.001; n = 15).

**Table 2:** Motor-function outcomes pre-treatment and at month 14 (n = 15).

		Baseline	Month 14	
		Median (range) [mean; SD]	Median (range) [mean; SD]	Change from baseline, median (range) [mean; SD]
MRC %	Total	71 (2.0–95.7) [67.3; 27.2]	77 (3.6–95.6) [68.2, 25.9]	+ 2 (− 11.8 to 11.4) [0.9; 6.8]
	Proximal	61 (0.7–98.7) [59.7; 28.8]	72 (3.3–95.3) [62.2; 27.0]	+ 5 (− 15.3 to 17.7) [2.5; 9.6]
	Distal	80 (2.9–96.2) [72.7; 27.4]	82 (3.8–97.1) [72.5; 25.8]	+ 1 (− 10.0 to 10.2) [− 0.2; 6.5]
	Upper limb	67 (3.1–96.4) [67.2; 25.9]	75 (4.3–96.4) [69.4; 24.3]	+ 1 (− 6.9 to 12.1) [2.2; 6.7]
	Proximal	59 (0.6–99.4) [61.4; 29.3]	71 (1.3–98.8) [65.6; 27.7]	+ 8 (− 13.1 to 15.6) [4.2; 9.3]
	Distal	72 (4.6–94.6) [70.8; 24.7]	76 (6.2–95.4) [71.8; 22.8]	+ 1 (− 10.0 to 12.7) [1.0; 7.1]
	Lower Limb	74 (0.4–97.5) [66.1; 30.9]	79 (0.7–95.0) [65.2; 29.7]	− 1 (− 20.7 to 10.4) [− 1.0; 8.2]
	Proximal	52 (0.8–97.5) [53.3; 30.3]	63 (1.7–93.3) [54.0; 28.4]	+ 1 (− 26.7 to 23.3) [0,7; 12.1]
	Distal	96 (0.0–100.0) [75.8; 34.4]	91 (0.0–100.0) [73.6; 32.6]	− 1 (− 16.3 to 8.8) [− 2.2; 7.3]

Change from baseline was calculated individually for every patient and is presented as the median change of all patients.

*MRC %* Medical Research Council Scale %, *SD* standard deviation.

### Muscle strength outcome in 15 treatment-naïve patients after 14 months of nusinersen therapy

Median total MRC % after 14 months of nusinersen treatment increased by 2%, reached 77% and ranged from 3.6 to 95.6% (Fig. 1b, Table 2). Six (40.0%) of fifteen patients had an increase in total MRC % (+ 5.1% to + 11.4%) above the cut-off, as predefined by ≥ 5% in the methods section. In contrast, another three patients (20.0%) demonstrated a relevant decline (− 6.4% to − 11.8%). Nearly half of the cohort (40.0%) remained stable in MRC scores (− 4.6 to + 4.0%). The three patients who showed a deterioration had baseline total MRC % values in the upper middle (66.8–85.4%), whereas the improving patients had scores ranging from 37.9 to 88.8%. Baseline MRC values of patients who remained stable were distributed across the full range (2.0–95.7%). All median MRC % changes of 72 tested muscles compared to baseline are provided in supplementary Table S5. The greatest changes (which were controlled for re-test reliability) were observed in arm abduction + 10% (MRC 55% at baseline) and shoulder external rotation + 10% (MRC 60% at baseline). These were followed by median MRC % changes of + 5% in arm elevation (MRC 45% at baseline), shoulder internal rotation (MRC 60% at baseline), elbow extension (MRC 60% at baseline), elbow flexion (MRC 65% at baseline), wrist flexion (MRC 85% at baseline) and thumb flexion (MRC 80% at baseline) (supplementary Table S5). A decline of 5% was observed in finger abduction (MRC 70% at baseline), and great toe extension (MRC 90% at baseline) (supplementary Table S5). No changes were observed in neck flexion and extension or shoulder elevation. The muscles identified as the weakest at baseline remained among the weakest tested muscles (Fig. 1b).

Interestingly, proximal muscle strength (+ 5%), in particular of the proximal upper limbs (+ 8%) including shoulder girdle function, showed the only relevant increase after 14 months of treatment with nusinersen. Proximal muscles of the lower limbs (+ 1%) including hip function did not reach the predefined cut-off change after 14 months of nusinersen treatment (Table 2).

### Longitudinal muscle strength evaluation in 15 treatment-naïve patients during treatment

Longitudinal changes of total muscle strength and predefined subdomains were further visualized in relation to the ability to walk. This important motor milestone was distributed equally in our cohort and distinguished between the weaker non-ambulatory and the stronger ambulatory patients.

**Figure 2:**
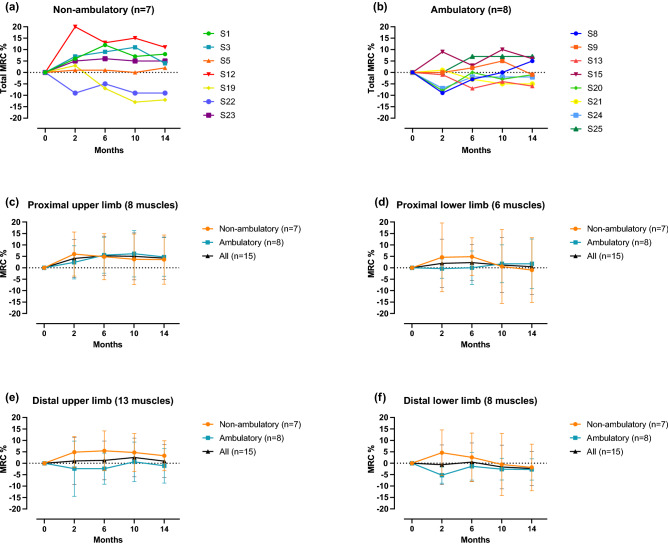
Median pre-post-comparisons to baseline (Δ) at assessed time points for total MRC % and subdomains during nusinersen therapy. Error bars indicate the interquartile range. (**a**,**b**) Individual trajectories of assessed patients for non-ambulatory (**a**) and ambulatory (**b**) patients showed a marked variability of treatment responses. The n-number indicates the number of analysed individuals. Patients’ pseudonyms are listed alongside the graphs (e.g. S1). (**c**–**f**) Depiction of MRC % changes in subdomains over time in ambulatory vs non-ambulatory patients and the overall cohort. Numbers in brackets indicate the number of muscle functions included in that specific subdomain. Improvements in both patient subgroups were observed in the proximal muscles of the upper limb (**c**). Muscle strength showed no relevant changes in the lower limbs with temporary increases above the threshold of non-ambulatory patients shortly after the loading dosing (**d**,**f**) regressing during further therapy. At month two distal muscle strength of ambulatory patients worsened (**e**,**f**), but the initial decline lessened over time.

In general, patients presented very individual trajectories of total MRC %. Muscle strength of four non-ambulatory patients increased by the end of nusinersen loading dosing at month two, followed either by a relatively stable period or a fading of previous improvements. Four of seven non-ambulatory patients had muscle strength improvements above the predefined cut-off during the observation period with one patient reaching increases up to 19%. However, two patients showed a decline in muscle strength despite ongoing therapy (Fig. 2a).

Individual total MRC % changes of ambulatory patients varied more widely during the treatment course, but with average changes below the cut-off of 5% at all time points (Fig. 2b). Fluctuations in total MRC % changes were especially present in patients with higher total MRC % at baseline (above 60%).

Of the predefined subdomains the proximal muscles of the upper limbs could be identified as the muscle group benefiting the most and continuously from nusinersen therapy in both subgroups. Non-ambulatory patients showed a relevant increase of muscle strength at month two, but were outperformed by ambulatory patients at month six (Fig. 2c). The proximal lower limb muscle strength showed no relevant changes at month 14 in both subgroups (Fig. 2d). Non-ambulatory patients presented an improvement just below the threshold by the end of loading dosing, which subsided again over time (Fig. 2d). In the distal muscles of the upper limbs non-ambulatory patients showed a more accumulating effect with therapy duration; however, overall improvement was weaker than in proximal muscles (Fig. 2e). In contrast, in the distal lower limb muscles of non-ambulatory patients a non-relevant decrease was detected, and yet again, the highest improvement from baseline was seen at the end of loading dosing (Fig. 2f). In ambulatory patients, distal muscle strength showed an initial decline, which lessened until month 14 of treatment, but remained present compared to baseline (Fig. 2e,f).

### Follow-up of seven adult SMA patients enrolled during ongoing nusinersen treatment

Seven SMA patients, of whom five (71%) were non-ambulatory, were enrolled during their ongoing nusinersen treatment (two at month 2, two at month 6 and three at month 10), and were followed up until 18–26 months of treatment. As inclusion time and therapy duration under nusinersen therapy individually vary in these seven patients, we chose only to describe total MRC % and MRC % of subdomains over time and not to compare individual muscle strength changes. Baseline characteristics are summarized in Table 1, outcome measures in supplementary Table S6.

**Figure 3:**
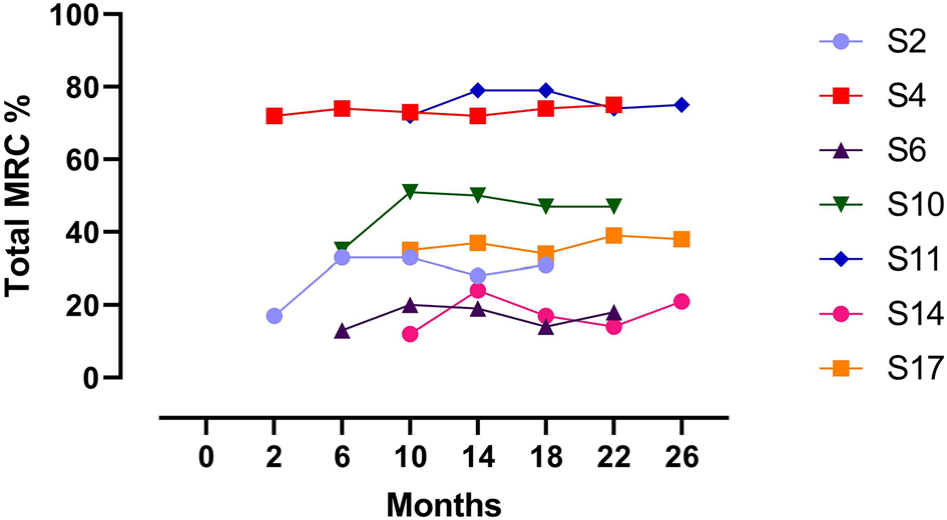
Individual MRC % trajectories of seven patients who were enrolled while ongoing nusinersen therapy. The first data point depicts the therapy month of enrolment; the last complies with the last assessment taken before data-cut. Individual variability of motor function in patients up to month 26 can be seen, with initial increases at the beginning of the observation period in five patients, succeeded by a predominantly stable period in three patients and a more variable trajectory in two patients. Comparing baseline to last assessment, improvement of motor function could be witnessed in most patients. Non-ambulatory patients presented with a total MRC % under 60% in the lower half of the graph.

Muscle strength fluctuated highly among individuals with an overall trend towards motor function improvement of the whole cohort and no further deterioration below the initially recorded total MRC % (Fig. 3). At data-cut (month 18–26 of nusinersen therapy) five out of the seven patients (S2, S6, S10, S14, S17) increased in total MRC % above the cut-off (+ 5.0 to + 15.3%) from the first assessment. These were all non-ambulatory, yet also demonstrated wider variation than the ambulatory patients (the two patients in the upper half of the graph). In four cases, the greatest muscle strength improvements were observed at the beginning of strength evaluation (month 6 to month 14 of therapy), followed by a plateau-like phase with fewer variations.

Similar to the 15 patients enrolled treatment-naïve, improvement of muscle strength was more prominent in proximal muscles. However, on average, proximal muscle strength improvement was comparable in the upper and lower limbs. In the distal upper limb muscles relevant improvements could be seen in five of the seven patients (+ 5.0 to + 18.1). On average, the smallest gain of muscle strength was observed in the distal lower limbs, though four non-ambulatory patients showed relevant increases (+ 5.6 to + 16.3) in strength (supplementary Table S6).

## Discussion

In this study, we observed an early gain of muscle strength in distinct body regions in adult SMA patients treated with nusinersen rather than an overall muscle strength improvement during 14 months of treatment. In particular, proximal upper limb and shoulder girdle muscles were earliest and most prominently improved by nusinersen, even in more advanced disease stages.

Thus far, treatment responses in adult SMA patients are commonly assessed by evaluating total scores of motor outcome measurements, such as the Hammersmith Functional Motor Scale Expanded (HFMSE) and the Revised Upper Limb Module (RULM), throughout the treatment course. Previously, these showed small but clinically relevant improvements over time^5–8^. The HFMSE score portrays the overall motor function, whereas RULM is meant to demonstrate the upper limb functionality^13,15^. Both, however, are not intended to describe specific motor function changes or to capture small muscle strength improvements. This raises questions about their potential to determine early treatment responses. Consistently, in the present study, when summarizing 72 muscles median muscle strength improvement (+ 2%) was small, but when looking at specific muscles, e.g. the proximal upper limb muscles (+ 8%), larger improvements could be observed. Thus, early changes might be missed when only assessing sum scores.

Although whole-body muscle strength increased only slightly, up to 80% of patients in our cohort enrolled treatment-naïve either increased or remained stable in total MRC %, which is not to be expected in the natural history^26^, thus a treatment-related effect is likely. Still, in 20% of treated patients, a decline was detected by summarizing whole-body muscle strength, raising the impression of an overall disease progression. However, this conclusion should be drawn with caution. Firstly, alike commonly used outcome measures, MMT is subject to floor and ceiling effects. Although its reliability rises with increasing numbers of tested muscles, it sinks with inclusion of strong muscles^27,28,32^. Here, patients with an identified decline were among the stronger ones in this cohort with total MRC % scores above 60%. Nevertheless, reliability was excellent in total MRC % and all subdomains. But in single muscle functions, wide variation from poor to excellent was seen. On average, ICCs values were highest in the proximal upper limbs and lowest in the distal lower limbs. Considering the minor disease involvement of distal muscles and the low ICC values, the observed decline in distal muscle strength of ambulatory patients could have been caused by ceiling effects. This, could have impacted the total MRC % towards a decline in overall muscle strength, skewing the real picture. Hence, changes of single muscle functions with high baseline MRC % (like trunk muscles and distal muscles) should be interpreted with caution.

Secondly, by looking at sum scores only, an improvement of specific muscles or function of clinical relevance might be missed. In all three patients declining in total MRC %, increases could be seen in at least seven muscle functions. Most of the improving muscle functions were located in the proximal upper limbs or the thumb. From patients' and caregivers' perspectives, minor improvements or even disease stabilisation are desirable and relevant for their individual quality of life and well-being. Especially in non-ambulatory patients small movements can have major impacts on quality of life. Thumb strength, in particular, can determine one’s mobility as it is needed in order to steer a powered wheelchair^16–18^. In this patient cohort all muscle functions of the thumb (range + 5 to + 15%) except for thumb abduction (+ 5% equal to its respective cut-off) showed a muscle strength gain.

To date, only little is known about specific regional features influencing muscle strength recovery by nusinersen. Recently, in a cohort of 256 non-ambulant pediatric SMA patients with types 2 and 3 higher improvements in muscle strength were observed in the upper limb measured by RULM in comparison to overall muscle strength as assessed by HFMSE after 38 months of nusinersen therapy^33^. Also, earlier recovery of smaller motor units of hand and forearm muscles was reported using motor unit number estimation investigation in children^34^. But no study intensively investigating differences in muscle strength improvement of specific muscles or body regions exist so far to the best of our knowledge. In our study, after 14 months of nusinersen treatment the strongest benefits were seen in proximal muscles of the upper limbs and shoulder girdle, followed by muscles of the forearm, hand and fingers and those with a preserved muscle strength. Very weak muscles were identified to improve less. Floor effects of MMT, loss of muscle fibers, replacement of muscle tissue by connective tissue and fat or fewer viable motor neurons could have limited the maximum attainable regain of strength^12^.

Restoring SMN protein by nusinersen might furthermore promote a length-dependent axonal regeneration of motor neurons leading to an earlier response of the shorter cervical motor neurons compared to the long motor neurons innervating the lower limbs^35^. With an increasing motor neuron length, higher concentrations of SMN protein may be needed to restore neuronal integrity and achieve consequent muscle strength improvements.

This hypothesis is supported by a distinct development of changes in muscle strength seen during the treatment course of non-ambulatory patients. Particularly in lower limb muscles the greatest improvement was observed shortly after loading dosing, which was fading as treatment intervals became longer during the maintenance dosing—similar to recently reported wearing-off phenomena^7,36^. Both, nusinersen dose and the duration of application intervals have been defined in accordance with studies in children under 12 years of age, and were not adjusted for age, weight or baseline motor function^3,4^. Further studies are needed to evaluate the efficiency of alternative doses or treatment regimens, as higher nusinersen doses or shorter intervals, and therefore higher SMN protein concentrations, might contribute to a globally distributed persistent muscle strength improvement.

Taking observed changes in the treatment course of the seven later enrolled patients of this study into account, another explanation needs to be considered: Comparable improvements of proximal upper and lower limb muscular strength were followed by relevant increases in the distal upper and the distal lower limb muscles in some patients. This suggests a time-dependent effect with a prolonged regeneration period of longer motor neurons. However, improvements of motor function from the first to the second evaluation, followed by a plateau-like phase, were observed in four of the seven patients at month 6–14 of nusinersen treatment—similar to the improvements seen during the earlier treatment course. Considering the variable time points, nusinersen hardly seems to be responsible for these observations. Here, fatigue after long enrollment procedures (approximately 60–90 min including MMT) with manual muscle testing scored last (20–25 min) and patients consequently scored lower on the first assessment might have had an influence. Naturally, this would also concern the witnessed changes in the treatment-naïve patients, particularly in the vulnerable non-ambulatory patients. Thus, an overestimation of actual treatment effects cannot be ruled out. However, considering the substantial amount of tested muscles (72 in total, 37 bilaterally) in this cohort, fatigue likely played a role in every muscle testing session, causing a generally lower scoring of patients throughout the observation period.

Moreover, three previous studies monitoring nusinersen therapy with MMT in SMA patients observed higher improvements in muscle strength. Measured on the MRC 0–5 scale, variable numbers of muscles/muscle regions (6, 14 and 16) were added up to summarized muscle strength scores^8–10^. After 10 to 14 months on nusinersen treatment, mild improvements in MRC sum scores were observed in all three studies. MRC sum scores of these studies can be transformed into MRC % according to the formula in the method section of this study^10,30^: hereby, mean MRC % increased by + 4.2% in the study of De Wel et al*.*^9^, + 2.8% in Walter et al*.*^8^, and + 2.5% in Moshe-Lilie et al*.*^10^, respectively. Compared to the mean MRC % increase of 0.9% in our study, here we observed a lower increase in overall muscle function. Next to fatigue, the selected muscle regions assessed in the described studies could have affected total scoring. While Moshe-Lilie et al*.* included more distal muscles, 7 of 16 (43.8%), De Wel et al*.* incorporated only 2 of 6 (33.3%) distal muscles. In our study 21 of 37 (56.8%) tested muscles were localized distally and were therefore more vulnerable to ceiling effects. Summarizing our MMT gradings of the muscles investigated in the study of De Wel et al*.*, a total mean MRC % change of 1.9% would have been detected—an increased, but still lower overall muscle strength score compared to the cohort of De Wel et al. Consequently, the fatigue experienced by our patients when testing 72 muscle functions might have rather led to the observation of smaller improvements than to an overestimation of treatment effects.

Therefore a more specific testing approach is preferable to (i) identify early treatment findings more precisely and (ii) avoid the potential impact of fatigue due to long testing periods.

We therefore suggest MMT of the proximal upper limb and shoulder girdle muscles as a valuable addition to the established motor function outcomes, especially in patients with advanced disease stages. Given that ongoing decline in muscle strength after treatment initiation was observed in 20% of our patients and with an alternative therapy now available, treatment non-responders need to be recognized as early as possible to prevent further motor neuron degeneration and to treat every patient with the individually best suitable drug^19^.

This study has some limitations: MMT scores are rated on a semiquantitative scale and are influenced by interrater- and retest-reliability, hence adjustments towards reliability analysis were performed by calculation of ICCs. Due to the ongoing pandemic, some data of month 14 had to be replaced, causing a potential over- or under-estimation of motor function changes between month 10 and 14. Considering the short observation period, the lack of an untreated control group and the small and heterogenous study cohort, we provide preliminary results of muscle strength changes during nusinersen treatment. To confirm and extrapolate our observations to other treatment modalities, larger studies using MMT along with objective assessments, such as dynamometry, motor unit number index (MUNIX), motor unit number estimation (MUNE) or magnetic resonance imaging, longer study duration and alternative treatments are needed.

In summary, we provide first data on distinct regional muscle strength improvements within 14 months of nusinersen treatment with the earliest recovery of muscle strength in the proximal upper limbs and shoulder girdle in adult SMA patients in different disease stages. Motor neuron length, motor unit size and residual muscle function might be good predictors for treatment benefit of predefined muscle groups. More specific motor outcome measures, rather than an overall motor function or strength measurement, should additionally be introduced in order to capture non-responders early in the treatment course.

## Supplementary Information

Supplementary Information.

## Data Availability

The data that support the findings of this study, especially the datasets generated during and/or analysed during the current study are available from the corresponding author on reasonable request.
