# Supplementary material for: Improvement of muscle strength in specific muscular regions in nusinersen-treated adult patients with 5q-spinal muscular atrophy
Source: Sci Rep. 2023 Apr 17;13:6240. doi: 10.1038/s41598-023-31617-5 (PMC10107562; doi:10.1038/s41598-023-31617-5)
Supplement: Supplementary file 1 — Supplementary Information. [file 41598_2023_31617_MOESM1_ESM.docx]

**Supporting Information**

**Improvement of muscle strength in specific muscular regions in nusinersen-treated adult patients with 5q-spinal muscular atrophy**

Olivia Schreiber-Katz^1°^, Hannah Alexandra Siegler^1°^, Gary Wieselmann^1^, Mareike Kumpe^1^, Gresa Ranxha^1^, Susanne Petri^1^, Alma Osmanovic^1,2*^

^1^ Department of Neurology, Hannover Medical School, 30625 Hannover, Germany; dr.schreiber-katz@t-online.de (O.S.-K.); Siegler.Hannah@mh-hannover.de (H.A.S.); Gary.Wieselmann@gmx.net (G.W.); mareikekumpe24@gmail.com (M.K.); Gresa.Ranxha1@gmail.com (G.R.); Petri.Susanne@mh-hannover.de (S.P.)

^2^  Essen Center for Rare Diseases (EZSE), University Hospital Essen, 45147 Essen, Germany

° These authors contributed equally to this work as first authors

Supplementary Table S1: Modified Medical research council scale

| **MRC Grade** | | **Definition** |
| --- | --- | --- |
| **0-5** | **0-10** |  |
| 5 | 10 | Normal strength |
| 5- | 9 | Barely detectable weakness |
| 4+ | 8 | Same as grade 4, but muscle holds the joint against moderate to maximal resistance |
| 4 | 7 | Muscle holds the joint against a combination of gravity and moderate resistance |
| 4- | 6 | Same as grade 4, but muscle holds the joint only against minimal resistance |
| 3+ | 5 | Muscle moves the joint fully against gravity and is capable of transient resistance, but collapses abruptly |
| 3 | 4 | Muscle cannot hold the joint against resistance, but moves the joint fully against gravity |
| 3- | 3 | Muscle moves the joint against gravity, but not through full mechanical range of motion |
| 2 | 2 | Muscle moves the joint fully when gravity is eliminated |
| 1 | 1 | A flicker of movement is seen or felt in the muscle |
| 0 | 0 | No movement |

**Supplementary Table S1:** Definition of MRC 0-10 grades and translation of scoring from MRC 0-5, adapted from Florence J.M. *et al*.: (Florence, J. M. *et al.*Intrarater reliability of manual muscle test (Medical Research Council scale) grades in Duchenne's muscular dystrophy. *Phys Ther* 72, 115-122; discussion 122-116, doi:10.1093/ptj/72.2.115 (1992). Abbreviations: MRC, Medical Research Council

Supplementary Table S2: Definition of MRC Items

| MRC % | Motor functions | | |
| --- | --- | --- | --- |
|  | Trunk | Upper limb | Lower limb |
| Total | neck flexion, neck extension | shoulder elevation, arm elevation, arm abduction, arm adduction, shoulder external rotation, shoulder internal rotation, elbow flexion, elbow extension, pronation, supination, wrist extension, wrist flexion, thumb flexion, thumb extension, thumb abduction, thumb adduction, thumb opposition, finger extension, finger flexion, finger abduction, abduction digiti minimi | hip flexion, hip extension, hip abduction, hip adduction, knee extension, knee flexion, foot dorsiflexion, foot plantarflexion, foot pronation, foot supination, great toe extension, great toe flexion, toe extension, toe flexion |
| Proximal | neck flexion, neck extension | shoulder elevation, arm elevation, arm abduction, arm adduction, shoulder external rotation, shoulder internal rotation, elbow flexion, elbow extension | hip flexion, hip extension, hip abduction, hip adduction, knee extension, knee flexion |
| Distal |  | pronation, supination, wrist extension, wrist flexion, thumb flexion, thumb extension, thumb abduction, thumb adduction, thumb opposition, finger extension, finger flexion, finger abduction, abduction digiti minimi | foot dorsiflexion, foot plantarflexion, foot pronation, foot supination, great toe extension, great toe flexion, toe extension, toe flexion |
| Upper limb |  | shoulder elevation, arm elevation, arm abduction, arm adduction, shoulder external rotation, shoulder internal rotation, elbow flexion, elbow extension, pronation, supination, wrist extension, wrist flexion, thumb flexion, thumb extension, thumb abduction, thumb adduction, thumb opposition, finger extension, finger flexion, finger abduction, abduction digiti minimi |  |
| Proximal |  | shoulder elevation, arm elevation, arm abduction, arm adduction, shoulder external rotation, shoulder internal rotation, elbow flexion, elbow extension |  |
| Distal |  | pronation, supination, wrist extension, wrist flexion, thumb flexion, thumb extension, thumb abduction, thumb adduction, thumb opposition, finger extension, finger flexion, finger abduction, abduction digiti minimi |  |
| Lower limb |  |  | hip flexion, hip extension, hip abduction, hip adduction, knee extension, knee flexion, foot dorsiflexion, foot plantarflexion, foot pronation, foot supination, great toe extension, great toe flexion, toe extension, toe flexion |
| Proximal |  |  | hip flexion, hip extension, hip abduction, hip adduction, knee extension, knee flexion |
| Distal |  |  | foot dorsiflexion, foot plantarflexion, foot pronation, foot supination, great toe extension, great toe flexion, toe extension, toe flexion |

**Supplementary Table S2**: Definition of MRC subdomains with the included motor functions of trunk, upper and lower limbs.

Supplementary Table S3: Intraclass Correlation Coefficients

| MRC % | ICC (95% confidence interval) | Maximum deviation of mean MRC % |
| --- | --- | --- |
|  |  |  |
| Total | 0.990 (0.974 – 0.997) | 2.2 |
| Proximal | 0.993 (0.983 – 0.998) | 2.3 |
| Distal | 0.981 (0.952 – 0.994) | 2.2 |
| Upper limb | 0.986 (0.961 – 0.995) | 2.1 |
| Proximal | 0.986 (0.964 – 0.996) | 2.2 |
| Distal | 0.977 (0.941 – 0.993) | 2.2 |
| Lower limb | 0.988 (0.969 – 0.996) | 2.3 |
| Proximal | 0.988 (0.969 – 0.996) | 1.7 |
| Distal | 0.974 (0.932 – 0.992) | 3.3 |
| Single muscle functions |  |  |
| Neck flexion | 0.926 (0.799 – 0.977) | 10.0 |
| Neck extension | 0.894 (0.727 – 0.967) | 7.5 |
| Shoulder elevation | 0.914 (0.774 – 0.973) | 4.6 |
| Arm elevation | 0.995 (0.987 – 0.998) | 3.3 |
| Arm abduction | 0.938 (0.840 – 0.980) | 8.3 |
| Arm adduction | 0.917 (0.784 – 0.974) | 5.4 |
| Shoulder external rotation | 0.988 (0.965 – 0.996) | 2.1 |
| Shoulder internal rotation | 0.893 (0.718 – 0.967) | 3.8 |
| Elbow flexion | 0.961 (0.900 – 0.988) | 1.7 |
| Elbow extension | 0.944 (0.851 – 0.982) | 2.1 |
| Pronation | 0.964 (0.906 – 0.989) | 1.7 |
| Supination | 0.965 (0.878 – 0.985) | 2.5 |
| Wrist extension | 0.975 (0.934 – 0.992) | 2.5 |
| Wrist flexion | 0.926 (0.808 – 0.977) | 2.5 |
| Thumb flexion | 0.885 (0.703 – 0.964) | 4.6 |
| Thumb extension | 0.906 (0.750 – 0.971) | 0.8 |
| Thumb abduction | 0.873 (0.674 – 0.960) | 5.0 |
| Thumb adduction | 0.932 (0.815 – 0.978) | 0.4 |
| Thumb opposition | 0.957 (0.880 – 0.987)* | 1.4 |
| Finger extension | 0.938 (0.828 – 0.982)* | 5.0 |
| Finger flexion | 0.944 (0.856 – 0.982) | 5.0 |
| Finger abduction | 0.926 (0.808 – 0.977) | 4.6 |
| Abduction digiti minimi | 0.937 (0.833 – 0.980) | 2.9 |
| Hip flexion | 0.962 (0.900 – 0.988) | 2.9 |
| Hip extension | 0.902 (0.748 – 0.969) | 8.7 |
| Hip abduction | 0.977 (0.949 – 0.993) | 6.3 |
| Hip adduction | 0.898 (0.732 – 0.968) | 5.8 |
| Knee extension | 0.991 (0.770 – 0.997) | 5.0 |
| Knee flexion | 0.921 (0.790 – 0.975) | 7.1 |
| Foot dorsiflexion | 0.776 (0.327 – 0.940)° | 3.5 |
| Foot plantarflexion | 0.907 (0.742 – 0.973)* | 2.3 |
| Foot pronation | 0.959 (0.861 – 0.985)* | 6.4 |
| Foot supination | 0.539 (-0.347 – 0.868)* | 3.2 |
| Great toe extension | 0.952 (0.871 – 0.986)* | 4.1 |
| Great toe flexion | 0.882 (0.681 – 0.965)* | 10.0 |
| Toe extension | 0.928 (0.805 – 0.979)* | 5.0 |
| Toe flexion | 0.940 (0.836 – 0.982)* | 7.3 |

**Supplementary Table S3**: Intraclass Correlation Coefficients (ICC) with their 95% confidence intervals and maximum deviation of the mean MRC % during the three tested time points (baseline, two weeks from baseline and four weeks from baseline; days 0, 14, and 21) of total MRC %, subdomains and single muscle functions (mean of both sides, if applicable). We used a mean-rating (k=3), absolute-agreement, 2-way mixed-effects model for ICC calculation and interpretation was performed by the guidelines published by Koo and Li^1^: ≥ 0.9 excellent; 0.9 – 0.75 good; 0.75 – 0.5 moderate or < 0.5 poor reliability. N-numbers were n=12, except when marked with * n=11 or ° n=10.
^1^ Koo, T.K.; Li, M.Y. A Guideline of Selecting and Reporting Intraclass Correlation Coefficients for Reliability Research. *Journal of chiropractic medicine* **2016**,
 *15*, 155-163, doi:10.1016/j.jcm.2016.02.012.

Supplementary Table S4: MRC % of 15 patients enrolled before nusinersen treatment at baseline and month 14

|  | | | | S1 | S3 | S5 | S8 | S9 | S12 | S13 | S15 | S19 | S20 | S21 | S22 | S23 | S24 | S25 |
| --- | --- | --- | --- | --- | --- | --- | --- | --- | --- | --- | --- | --- | --- | --- | --- | --- | --- | --- |
| SMA type | | | | 3 | 2 | 2 | 3 | 3 | 2 | 3 | 3 | 2 | 3 | 3 | 3 | 2 | 4 | 3 |
| Ambulatory | | | | no | no | no | yes | yes | no | yes | yes | no | yes | yes | no | no | yes | yes |
| MRC % | **Total** | | Baseline | 66.5 | 34.6 | 2.0 | 78.6 | 81.4 | 37.9 | 85.4 | 70.6 | 66.8 | 95.6 | 95.7 | 67.2 | 42.9 | 95.6 | 88.8 |
|  |  |  | Month 14 | 74.9 | 38.6 | 3.6 | 83.8 | 79.7 | 49.3 | 79.0 | 76.9 | 55.0 | 95.1 | 91.1 | 57.8 | 48.3 | 94.0 | 95.6 |
|  | **Proximal** | | Baseline | 47.3 | 33.3 | 0.7 | 54.0 | 65.3 | 26.7 | 78.3 | 64.0 | 57.3 | 94.7 | 98.7 | 60.7 | 30.3 | 95.7 | 88.7 |
|  |  |  | Month 14 | 59.3 | 39.0 | 3.3 | 71.7 | 75.3 | 39.7 | 74.7 | 72.0 | 42.0 | 92.3 | 90.0 | 48.3 | 35.0 | 94.7 | 95.3 |
|  | **Distal** | | Baseline | 80.2 | 35.5 | 2.9 | 96.2 | 92.9 | 46.0 | 90.5 | 75.2 | 73.6 | 96.2 | 93.6 | 71.9 | 51.9 | 95.5 | 88.8 |
|  |  |  | Month 14 | 86.0 | 38.3 | 3.8 | 92.4 | 82.9 | 56.2 | 82.1 | 80.5 | 64.3 | 97.1 | 91.9 | 64.5 | 57.9 | 93.6 | 95.7 |
|  | **Upper limb** | | Baseline | 63.8 | 50.7 | 3.1 | 80.5 | 80.7 | 37.9 | 83.3 | 61.0 | 66.4 | 96.4 | 94.3 | 67.1 | 41.0 | 94.5 | 87.9 |
|  |  |  | Month 14 | 75.2 | 55.2 | 4.3 | 83.6 | 79.5 | 50.0 | 77.4 | 72.9 | 60.2 | 96.4 | 90.0 | 60.2 | 47.6 | 93.1 | 96.0 |
|  |  | **Proximal** | Baseline | 53.1 | 44.4 | 0.6 | 58.8 | 68.1 | 24.4 | 80.0 | 58.8 | 56.9 | 99.4 | 99.4 | 66.3 | 25.6 | 95.6 | 90.0 |
|  |  |  | Month 14 | 68.1 | 52.5 | 1.3 | 74.4 | 81.3 | 35.6 | 79.4 | 70.6 | 48.8 | 98.1 | 91.3 | 53.1 | 36.3 | 95.0 | 98.8 |
|  |  | **Distal** | Baseline | 70.4 | 54.6 | 4.6 | 93.8 | 88.5 | 46.2 | 85.4 | 62.3 | 72.3 | 94.6 | 91.2 | 67.7 | 50.4 | 93.8 | 86.5 |
|  |  |  | Month 14 | 79.6 | 56.9 | 6.2 | 89.2 | 78.5 | 58.8 | 76.2 | 74.2 | 67.3 | 95.4 | 89.2 | 64.6 | 54.6 | 91.9 | 94.2 |
|  | **Lower limb** | | Baseline | 68.9 | 7.1 | 0.4 | 74.3 | 81.1 | 36.8 | 87.5 | 82.9 | 65.4 | 93.9 | 97.5 | 65.4 | 45.0 | 96.8 | 89.3 |
|  |  |  | Month 14 | 72.5 | 12.5 | 0.7 | 82.9 | 78.9 | 47.1 | 80.4 | 81.4 | 44.6 | 92.9 | 92.1 | 52.9 | 49.3 | 95.0 | 94.6 |
|  |  | **Proximal** | Baseline | 32.5 | 10.8 | 0.8 | 40.0 | 55.8 | 25.0 | 72.5 | 65.0 | 51.7 | 87.5 | 97.5 | 47.5 | 32.5 | 95.0 | 85.0 |
|  |  |  | Month 14 | 40.8 | 18.3 | 1.7 | 63.3 | 64.2 | 40.8 | 65.0 | 69.2 | 25.0 | 83.3 | 86.7 | 37.5 | 30.8 | 93.3 | 90.0 |
|  |  | **Distal** | Baseline | 96.3 | 4.4 | 0.0 | 100.0 | 100.0 | 45.6 | 98.8 | 96.3 | 75.6 | 98.8 | 97.5 | 78.8 | 54.4 | 98.1 | 92.5 |
|  |  |  | Month 14 | 96.3 | 8.1 | 0.0 | 97.5 | 90.0 | 51.9 | 91.9 | 90.6 | 59.4 | 100.0 | 96.3 | 64.4 | 63.1 | 96.3 | 98.1 |

**Supplementary Table S4**: Individual baseline and month 14 values of total MRC % and subdomains in SMA patients who were enrolled before nusinersen treatment.

Supplementary Table S5: Pre-treatment and month 14 change of single muscle functions

| MRC % | | Pre-treatment | Month 14 |
| --- | --- | --- | --- |
|  |  |  | Median change |
|  |  | Median (range) | Median (range) |
| Trunk  muscles | Neck flexion | 100 (30-100) | 0 (-30-30) |
|  | Neck extension | 100 (60-100) | 0 (-10-30) |
| Upper limb | Shoulder elevation | 100 (0-100) | 0 (-30-35) |
|  | Arm elevation | 45 (0-100) | +5 (-30-35)* |
|  | Arm abduction | 55 (5-100) | +10 (-25-45)* |
|  | Arm adduction | 70 (0-100) | 0 (-20-40) |
|  | Shoulder external rotation | 60 (0-100) | +10 (-15-35)* |
|  | Shoulder internal rotation | 60 (0-100) | +5 (-45-30)* |
|  | Elbow flexion | 65 (0-100) | +5 (-15-25)* |
|  | Elbow extension | 60 (0-100) | +5 (-25-30)* |
|  | Pronation | 80 (0-100) | 0 (-15-15) |
|  | Supination | 80 (0-100) | 0 (-20-25) |
|  | Wrist extension | 90 (0-100) | 0 (-15-15) |
|  | Wrist flexion | 85 (0-100) | +5 (-25-15)* |
|  | Thumb extension | 75 (0-100) | 0 (-15-15) |
|  | Thumb flexion | 80 (0-100) | +5 (-40-25)* |
|  | Thumb abduction | 70 (0-85) | +5 (-40-25) |
|  | Thumb adduction | 75 (40-100) | 0 (-25-20) |
|  | Thumb opposition | 70 (5-100) | 0 (-20-25) |
|  | Finger extension | 75 (0-100) | 0 (-10-10) |
|  | Finger flexion | 80 (10-100) | 0 (-15-25) |
|  | Finger abduction | 70 (5-100) | -5 (-45-30)* |
|  | Abduction digiti minimi | 70 (0-100) | 0 (-20-30) |
| Lower limb | Hip flexion | 20 (0-90) | 0 (-20-20) |
|  | Hip extension | 60 (0-95) | +5 (-15-60) |
|  | Hip abduction | 90 (0-100) | 0 (70-25) |
|  | Hip adduction | 70 (5-100) | 0 (-55-40) |
|  | Knee extension | 30 (0-100) | 0 (-20-30) |
|  | Knee flexion | 70 (0-100) | -5 (-25-40) |
|  | Foot dorsiflexion | 85 (0-100) | 0 (-25-20) |
|  | Foot plantarflexion | 100 (0-100) | 0 (-20-10) |
|  | Foot pronation | 100 (0-100) | 0 (-25-40) |
|  | Foot supination | 100 (0-100) | 0 (-50-35) |
|  | Great toe extension | 90 (0-100) | -5 (-25-30)* |
|  | Great toe flexion | 100 (0-100) | -5 (-50-20) |
|  | Toe extension | 90 (0-100) | -5 (-30-25) |
|  | Toe flexion | 100 (0-100) | 0 (-20-20) |

**Supplementary Table S5:** Pre-treatment values and change at month 14 of single muscle functions (mean of both sides, if applicable). Change at month 14 was calculated individually for every patient and presented as the median change. Actual changes above the corresponding cut-offs (maximum observed deviation of mean MRC %) are highlighted with *.

Supplementary Table S6: Seven patients enrolled during ongoing nusinersen treatment

|  | | | | S2 | S4 | S6 | S10 | S11 | S14 | S17 |
| --- | --- | --- | --- | --- | --- | --- | --- | --- | --- | --- |
| First assessment - month | | | | 2 | 2 | 6 | 6 | 10 | 10 | 10 |
| Last assessment - month | | | | 18 | 22 | 22 | 22 | 26 | 26 | 26 |
| SMA type | | | | 2 | 3 | 2 | 3 | 3 | 2 | 2 |
| Ambulatory | | | | no | yes | no | no | yes | no | no |
| MRC % | **Total** | | Baseline | 17.1 | 74.4 | 13.9 | 36.3 | 75.0 | 12.6 | 35.1 |
|  |  |  | Outcome | 32.4 | 78.1 | 18.9 | 48.5 | 78.9 | 21.1 | 40.7 |
|  | **Proximal** | | Baseline | 6.3 | 63.3 | 11.3 | 32.0 | 60.7 | 12.3 | 28.3 |
|  |  |  | Outcome | 26.7 | 70.7 | 18.3 | 39.3 | 70.3 | 18.0 | 38.0 |
|  | **Distal** | | Baseline | 24.8 | 82.4 | 15.7 | 39.3 | 85.2 | 12.9 | 40.9 |
|  |  |  | Outcome | 36.4 | 83.3 | 19.3 | 55.0 | 85.0 | 23.3 | 42.6 |
|  | **Upper limb** | | Baseline | 24.3 | 70.0 | 15.5 | 41.0 | 68.1 | 16.2 | 40.8 |
|  |  |  | Outcome | 39.3 | 71.9 | 20.0 | 55.0 | 74.0 | 25.2 | 48.1 |
|  |  | **Proximal** | Baseline | 6.3 | 67.5 | 10.6 | 40.0 | 55.0 | 10.6 | 34.4 |
|  |  |  | Outcome | 31.3 | 70.0 | 18.8 | 47.5 | 62.5 | 16.3 | 43.8 |
|  |  | **Distal** | Baseline | 35.4 | 71.5 | 18.5 | 41.5 | 76.2 | 19.6 | 45.5 |
|  |  |  | Outcome | 44.2 | 73.1 | 20.8 | 59.6 | 81.2 | 30.8 | 50.8 |
|  | **Lower limb** | | Baseline | 6.8 | 79.3 | 9.6 | 25.7 | 83.6 | 4.3 | 26.0 |
|  |  |  | Outcome | 20.0 | 85.7 | 15.0 | 36.1 | 84.6 | 12.1 | 28.2 |
|  |  | **Proximal** | Baseline | 5.8 | 51.7 | 7.5 | 12.5 | 61.7 | 7.5 | 18.3 |
|  |  |  | Outcome | 15.0 | 66.7 | 12.5 | 20.8 | 75.8 | 13.3 | 26.7 |
|  |  | **Distal** | Baseline | 7.5 | 100.0 | 11.3 | 35.6 | 100.0 | 1.9 | 33.1 |
|  |  |  | Outcome | 23.8 | 100.0 | 16.9 | 47.5 | 91.3 | 11.3 | 29.4 |

**Supplementary Table S6**: Time of first and last assessment in months and individual baseline and outcome MRC % values of SMA patients who were enrolled during ongoing treatment. Baseline was defined as the first documented value and outcome as the last one.
